# Monitoring Damage Propagation in Glass Fiber Composites Using Carbon Nanofibers

**DOI:** 10.3390/nano6090169

**Published:** 2016-09-10

**Authors:** Ahmed Al-Sabagh, Eman Taha, Usama Kandil, Gamal-Abdelnaser Nasr, Mahmoud Reda Taha

**Affiliations:** 1 Egyptian Petroleum Research Institute, Nasr City, Cairo 11727, Egypt; alsabaghh@gmail.com (A.A.-S.); eman@unm.edu (E.T.); alfa_olefins@yahoo.com (U.K.); 2 Department of Physics, Faculty of Science, Cairo University, Giza 12613, Egypt; rrrrrgmal@yahoo.com; 3 Department of Civil Engineering, University of New Mexico, Albuquerque, NM 87131, USA

**Keywords:** carbon nanofibers, glass fiber composites, self-sensing, damage monitoring

## Abstract

In this work, we report the potential use of novel carbon nanofibers (CNFs), dispersed during fabrication of glass fiber composites to monitor damage propagation under static loading. The use of CNFs enables a transformation of the typically non-conductive glass fiber composites into new fiber composites with appreciable electrical conductivity. The percolation limit of CNFs/epoxy nanocomposites was first quantified. The electromechanical responses of glass fiber composites fabricated using CNFs/epoxy nanocomposite were examined under static tension loads. The experimental observations showed a nonlinear change of electrical conductivity of glass fiber composites incorporating CNFs versus the stress level under static load. Microstructural investigations proved the ability of CNFs to alter the polymer matrix and to produce a new polymer nanocomposite with a connected nanofiber network with improved electrical properties and different mechanical properties compared with the neat epoxy. It is concluded that incorporating CNFs during fabrication of glass fiber composites can provide an innovative means of self-sensing that will allow damage propagation to be monitored in glass fiber composites.

## 1. Introduction

In recent years, textile fabric composites have attracted widespread attention owing to their superior properties, making them attractive materials for many applications like smart fabrics and the aerospace, marine, and automobile industries [1,2]. Glass fiber textile composites, in particular, are being widely researched currently due to their relatively high strength-to-weight ratio at low cost. The properties of glass fiber textile-reinforced polymer composites greatly depend on the fabric structure as well as the matrix properties. Many attempts have been made to improve their properties for high performance textile fabric composites. Several studies reported considerable improvement in fracture toughness of glass fiber reinforced polymer (GFRP) when fumed silica, carbon black, carbon nanotubes (CNTs) [3] and carbon nanofibers (CNFs) [4] were incorporated in polymer nanocomposites. The tensile strength of GFRP was improved using carbon nanofibers [5] and CNTs [6]. Significant enhancements in thermal and electrical conductivities of GFRP with carbon nanomaterials were reported [7,8,9].

In this sense, nanomaterials play an important and significant role in controlling the intrinsic properties of glass fiber composites. Carbonaceous nanomaterials such as CNTs and CNFs are the most common and the most promising additives used to fabricate multifunctional glass textile nanocomposites with enhanced capabilities. For instance, CNFs are attractive candidates in conductivity-related applications because of their relatively high electrical conductivity [10]. A percolated concentration must be incorporated to transfer the composite from an insulate state into conductive state, which is known as the percolation threshold. At this concentration, the conductivity is improved by several orders of magnitude and percolation network is achieved between conductive particles [11]. Yang et al. [12] found that the electrical conductivity of CNFs-filled polystyrene (PS) composite improved by ten orders of magnitude over that of the neat PS with percolation threshold 4 wt %, providing high electromagnetic interference shielding (EMI) at low filler loading. Yang at el. [13] confirmed the percolation threshold of CNFs to be 5 wt % with a decrease in electrical resistivity by 11 orders of magnitude. Lozano at el. [14] examined the electrical behavior of CNFs-filled polypropylene (PP) for electrostatic dissipation (ESD) applications, and the percolation was found to be 9–18 wt %. It was also found that the percolation threshold of CNFs/polypropylene (PP) composites was about 3–5 vol % depending on the degree of graphitization (graphite perfection) of CNFs [15,16]. Despite the fact that CNFs significantly improve the electrical conductivity of polymers, there has been very limited research using CNFs for self-sensing applications. Park et al. [17] found that CNFs/epoxy composites have reliable self-sensing capability under both static and cyclic loading conditions. The aspect ratio of CNFs was reported to have significant effects in the formation of electrically percolated networks. In addition, prior works [18,19] have confirmed that damage monitoring using conductive CNFs composites is strongly dependent on forming a three-dimensional conductive network inside the polymer matrix. 

To date, the ability of CNFs in monitoring damage propagation in glass textile-reinforced polymer composites has not yet been reported. In this study, the ability of CNFs to improve electrical conductivity and alter the mechanical properties of epoxy nanocomposites is investigated. The conductive CNFs/epoxy nanocomposite is then used to monitor damage propagation in glass textile-reinforced polymer composites under static loading.

## 2. Materials and Methods

### 2.1. Materials and Fabrication

CNFs were supplied by Nanostructured & Amorphous Materials Inc. They had diameter of 80–200 nm and a length of 0.5-20 µm and thus an aspect ratio ranging between 6.3 and 100. The epoxy used in fabrication was EPOTUF^®^ 37-127 epoxy system supplied by U.S. Composites, Inc. (West Palm Beach, FL, USA) The epoxy resin is low viscosity, 100% reactive diluted liquid based on Bisphenol-A containing glycidyl ether. The hardener was Aliphatic Amine EPOTUF^®^ 37-614. The resin to hardener mixing ratio was 2:1. The bidirectional S-Glass fiber fabric was supplied by ACP Composites, Inc. (Livermore, CA, USA).

The performance of the CNFs-polymer nanocomposite is affected by the homogeneity of nanofibers dispersion into the polymer matrix. Several dispersion methods were recommended in the literature to obtain homogeneously dispersed CNFs in the polymer matrix and to avoid agglomeration. CNFs with different contents (0, 0.3, 0.5, 1.0, 1.5, 2.0 and 2.5 wt %) were first hand-stirred into the epoxy resin, and then sonicated in a path sonicator for 1 h at 40 °C and frequency of 40 kHz. The resin-CNFs mixture was further dispersed using a high shear mixer at speed 11,000 rpm at a temperature of 90 °C for 1 h. The resin-CNFs mixture was then mechanically stirred at temperature of 90 °C for 2 h and a speed of 800 rpm. The resin-CNFs mixture was degassed to remove the bubbles for 30 min at 50 °C and then left to cool for 1 h at room temperature. After cooling, the epoxy hardener was hand-stirred into the resin-CNFs mixture for 5 min and left overnight. CNFs/epoxy nanocomposite was then cured for 2.5 days at 110 °C to ensure full curing. 

**Figure 1:**
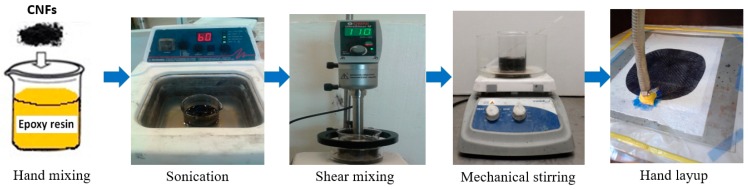
Schematic representation of glass fiber composite fabrication incorporating CNFs.

To prepare glass fiber reinforced (GFRP) composites, after adding the epoxy hardener to the resin-CNFs mixture, that mixture was then used to fabricate the GFRP using the hand layup technique. Six layers of bidirectional plain weave glass fiber textile fabrics were laid in 0° direction, and then vacuum pressure was applied for 24 h. The glass fiber composite plates were then cured for 2.5 days at 110 °C to insure complete curing. 2 wt % CNFs were used to fabricate glass fiber composites. The CNFs content used for producing the glass fiber composites was based on the electrical percolation observations of epoxy-CNFs nanocomposites discussed below. Figure 1 presents schematically the glass fiber composite fabrication method. Fiber volume fraction of glass fiber composites incorporating 2.0 wt % CNFs was determined using ASTM D3171 [20] and was found to be 55.6%. To examine the dispersion of CNFs in the epoxy matrix, fractured surfaces of the epoxy-CNFs nanocomposites were covered with a layer of gold and were then investigated using the Field Emission Scanning Electron Microscope (FESEM) using Quanta 250, FEI Company’s Quanta 250. Fourier transform infrared (FTIR) spectra was also recorded using Nicolet IS-10 FTIR spectrophotometer-Thermo Fisher Scientific within 400–4000 cm^−1^ wave number.

### 2.2. Electrical and Mechanical Measurements of the Composites

**Figure 2:**
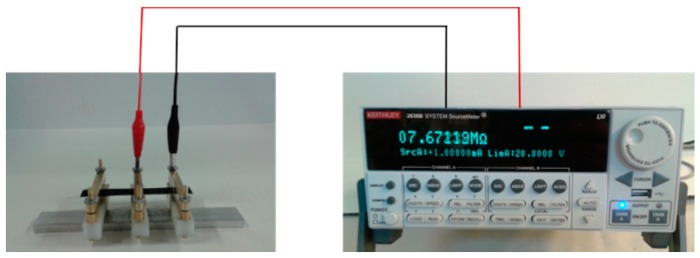
The strip electrode used to determine the electrical resistance of CNFs/epoxy nanocomposites.

The electrical conductivity of CNFs/epoxy polymer nanocomposites was determined according to ASTM D257 [21]. Measurements were performed using a Keithley 2636b source meter and strip electrodes via a standard two-probe configuration as shown in Figure 2. 

Silver paint was used to ensure good contact between the specimens and the electrode. The electrical conductivity (σ) was calculated using Equation (1).

(1) $$\sigma =\;\frac{L}{AR}$$

*A* is the cross sectional area, *L* is the length, and *R* is the measured electrical resistance.

To identify the significance of CNF on the elastic properties of CNFs/epoxy nanocomposites, three specimens of 20 mm × 10 mm × 3 mm were tested. Dynamic mechanic analysis (DMA) was performed on a Triton Instruments, operating in the tension mode at an oscillation frequency of 1 Hz. Data were collected from room temperature to 150 °C at a scanning rate of 10 °C/min. For static tension properties of CNFs/epoxy nanocomposites, three specimens of 20 mm × 10 mm × 2 mm were tested using Triton Instruments, operating in the static tension mode with preload force 0.01 N, load rate 0.2 N/min and to maximum force 2 N.

**Figure 3:**
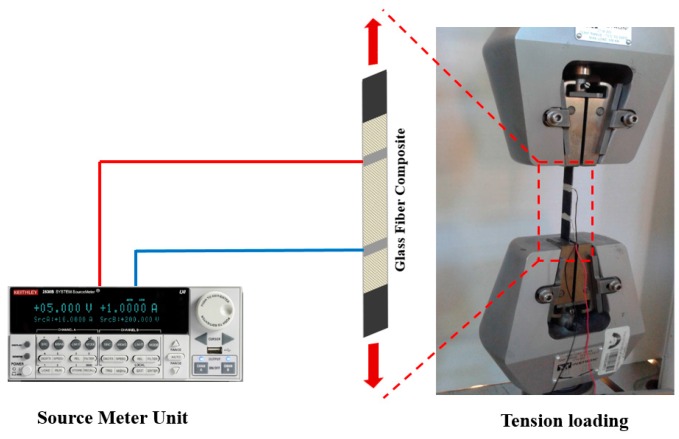
Schematic of electrical resistance measurement of glass fiber composite coupons during tension tests.

Three GFRP composite coupons of 19 mm × 150 mm were tested under off-axis (i.e., load was applied at 45° with respect to the fiber direction) static monotonically increasing tension stress. The reason for choosing off-axis loading was to simulate realistic loading conditions where stresses are generated in any direction to the glass fiber composite. It is also well established that behavior of the fiber composites in the off-axis direction is governed by the polymer matrix rather than the fibers [6]. Therefore, off-axis tension would best show the significance of CNFs. The static tension tests were performed using MTS^®^ Bionex servo hydraulic machine. A displacement control protocol was used in the static tension tests according to the ASTM standards methods D3039/D3039M [22] with a loading rate of 1.0 mm/min. The electrical resistance of the glass fiber composite specimens was measured during the tension test using a Keithley 2636B source meter. Conductive electrodes were applied at the glass fiber composite coupon using silver paint at two points spaced by 50 mm to allow electric resistance measurements. Schematic representation of the electrical resistance measurement during the static monotonically increasing tension test is shown in Figure 3. 

Damage in glass fiber composite coupons was estimated in terms of the change of the electrical conductivity during loading. The electrical conductivity was measured and a metric of damage based on electrical conductivity change denoted *D_E_*(*t*) was calculated using Equation (2).

(2) $$D_{E}\left(t\right)=\;1-\;\frac{\sigma \left(t\right)}{\sigma \left(t_{0}\right)}\;\%$$

where *D_E_*(*t*) is the electrical damage measured at time *t*, σ(*t*_0_) is the initial electrical conductivity of the composite prior to load application at time *t_0_*, and σ(*t*) is the electrical conductivity of the composite at time *t*. Moreover, a metric of damage based on change of modulus of elasticity, representing mechanical damage and denoted *D_M_*(*t*) was calculated using Equation (3):

(3) $$D_{M}\left(t\right)=\;1-\;\frac{E\left(t\right)}{E\left(t_{0}\right)}\;\%$$

where *E*(*t_0_*) is the initial tangent modulus of elasticity of the glass fiber composite coupon at *t_0_* and *E*(*t*) is the tangent modulus of elasticity of the glass fiber composite coupon at time *t*. A minimum tangent modulus of zero was assumed to account for the descending stress-strain.

## 3. Results and Discussion

### 3.1. Mechanical Properties of CNFs/Epoxy Nanocomposites

**Figure 4:**
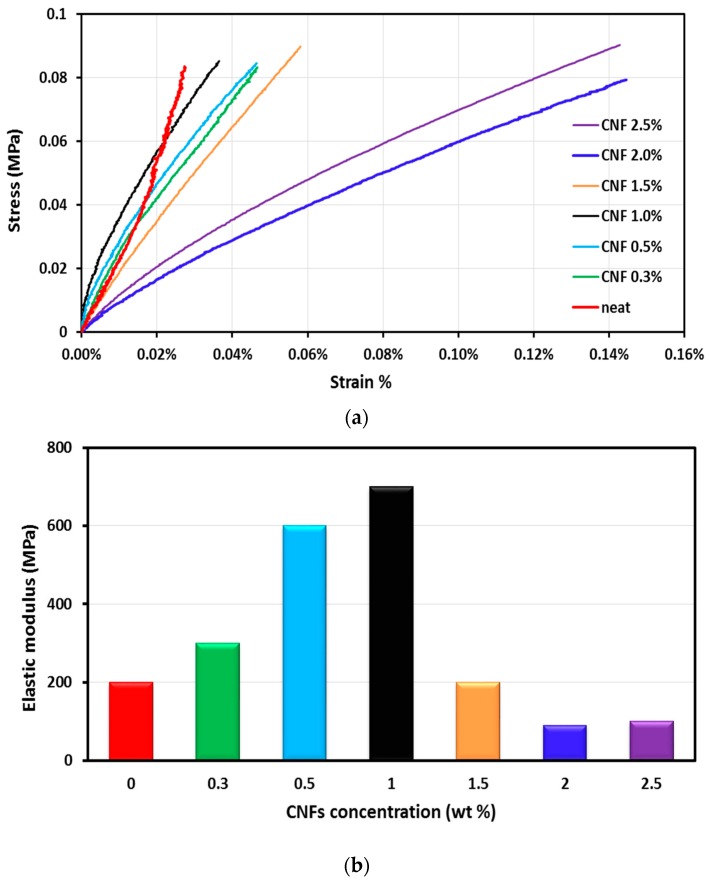
(**a**) The stress-strain relationship for CNFs/epoxy nanocomposites; and (**b**) the change in elastic modulus versus the CNFs concentrations for CNFs/epoxy nanocomposites.

The mechanical properties of CNFs/epoxy nanocomposites can be observed from a static tension test, shown in Figure 4a,b. The stress-strain relationship observed for CNFs/epoxy nanocomposites at room temperature and the change in elastic modulus (*E*) versus the CNFs concentrations are shown in Figure 4a,b respectively. The results show strong influence of the presence of the CNFs on the mechanical properties of CNFs/epoxy nanocomposites, a gradual increase of material stiffness by increasing the amount of CNFs within the epoxy matrix up to 1.0 wt % of CNFs and then a sharp decrease in the material stiffness for composite filled 1.5 wt %, 2.0 wt % and 2.5 wt % CNFs. Moreover, the elastic modulus increases from 200 MPa to 700 MPa with increasing the CNFs concentrations from 0 to 1.0 wt % and drops to 200 MPa at 1.5 wt % CNFs, to 90 MPa at 2.0 wt % CNFs and to 100 MPa at 2.5 wt %. The reduction of elastic modulus at 2.0 wt % and 2.5 wt % CNFs/epoxy composite could be attributed to the effect of CNFs on the epoxy network. We suggest that high content of CNFs reduced the crosslinking degree of the epoxy matrix. Further in-depth investigations were conducted below to examine this hypothesis.

To better understand the significance of fillers/fibers on the mechanical properties of polymer composites, a number of models exist in the literature [23,24,25]. We further examine here the model by Christensen [24] to predict the elastic modulus of CNFs/epoxy nanocomposites:

(4) $$E_{c}=\;\frac{V_{f}}{6}\;E_{f}+\;\left[\frac{1}{1-V_{f}}\;\left(1+\frac{V_{f}}{4}+\;\frac{{V_{f}}^{2}}{6}\right)E_{m}\right]$$

where *E_c_*, *E_m_* and *E_f_* are the moduli of composite, matrix and filler respectively and *V_f_* is the filler/fiber volume fraction.

**Figure 5:**
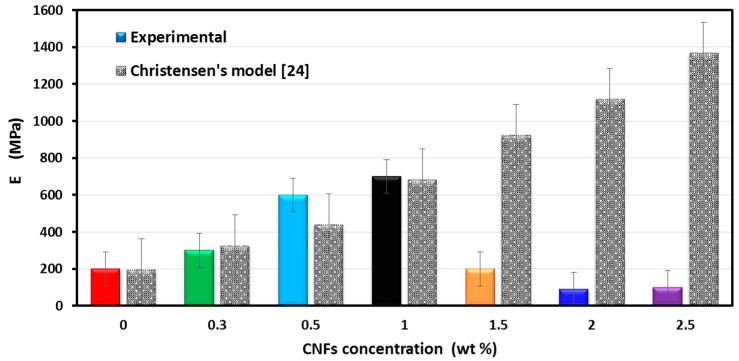
The experimental and predicted values of Young’s modulus of CNFs/epoxy nanocomposites.

Figure 5 compares the experimentally measured Young’s modulus of CNFs/epoxy nanocomposites with the values predicted using Christensen’s model [25]. The predicted values given by Christensen’s model showed a good agreement with the experimental observation of *E_c_* except at CNFs contents above 1.0 wt % CNFs. At low CNFs contents, the significance of the CNFs on the epoxy matrix is negligible, and thus CNFs work as fibers that reinforce the epoxy matrix and improve its mechanical properties as a composite. This behavior follows classical composite theory and thus good agreement with the model can be observed. Conversely, at 2.0 wt % and 2.5 wt % CNFs (i.e., higher CNFs concentrations), CNFs start to induce a chemical effect on the epoxy matrix, hindering the reaction between the resin and the hardener and affecting epoxy crosslinking. This adversely affects the mechanical properties of CNFs/epoxy nanocomposite. The effect of CNFs on epoxy crosslinking is not considered in Christensen’s model [25] and all other composite models in the literature, which only account for the reinforcing effect of the fillers. This model therefore failed to predict the modulus of elasticity of CNFs/epoxy nanocomposite incorporating high CNFs content as shown in Figure 5. Interestingly, composite filled with 1.5 wt % CNFs (at the transition between low and high CNFs contents) has no positive or negative effect on the elastic modulus. The 1.5 wt % CNFs is not high enough to chemically affect epoxy crosslinking. In addition, this concentration failed to act as reinforcing filler, having no effect on the elastic modulus of the epoxy nanocomposite.

**Figure 6:**
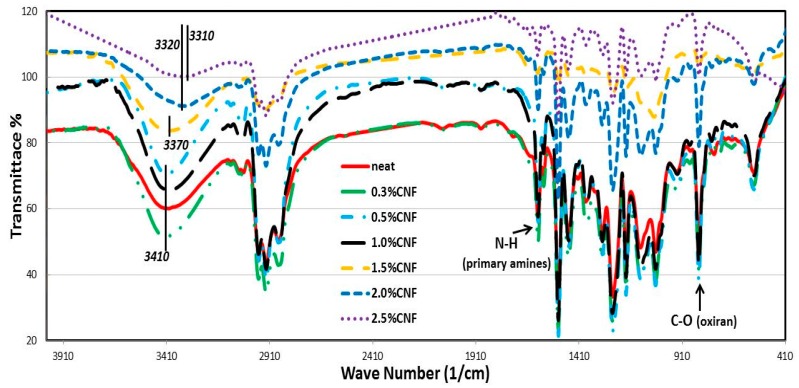
FTIR spectra of neat epoxy and CNFs/epoxy nanocomposites.

To further confirm the above hypothesis of interaction between the CNFs and epoxy and the chemical significance of high CNFs on epoxy, FTIR analysis was performed. FTIR is utilized to observe chemical changes of the epoxy matrix after incorporating CNFs. The FTIR spectra of the epoxy matrix with and without CNFs is presented in Figure 6. Figure 6 shows the absorption bands corresponding to C–H (2800–2970 cm^−1^), epoxide ring (~830 cm^−1^), N–H of primary amines (1590–1640 cm^−1^), O–H groups (3200–3600 cm^−1^), C–N (1040–1120 cm^−1^) and ether (~1250 cm^−1^). 

Comparing the FTIR spectrum of 2.0 wt % CNFs/epoxy with other spectra of CNFs/epoxy polymer nanocomposites and the neat epoxy, it can be observed that incorporating 2.0 wt % CNFs in the epoxy matrix caused an increase in the epoxide ring and primary N–H band intensity. In addition, the band of the hydroxyl group is significantly shifted to a lower intensity and to a lower wave number value (3410–3320 cm^−1^) at this wt % of CNFs compared with other wt %. It is obvious from the FTIR spectra in Figure 6 that for the neat and low concentration CNFs samples (0.3, 0.5 and 1.0 wt %) the O–H peak appeared almost around 3410 cm^−1^. This is attributed to the fact that at such low CNFs concentrations, there is a minor effect from CNFs on epoxy curing. Incorporating CNFs concentrations of 1.5, 2.0 and 2.5 wt % into the epoxy matrix appeared to shift the O–H peak to 3370 cm^−1^, 3320 cm^−1^ and 3310 cm^−1^, respectively. This may be attributed to the effect of CNFs on the curing behavior of the epoxy matrix [26]. It is well known that the broad complex band of the hydroxyl stretching vibration region at about 3200–3600 cm^−1^ is attributed to the combined effect of the differently associated hydroxyl groups, i.e., hydrogen bonding between hydroxyl and hydroxyl/carbonyl groups of different strength and hydrogen bonding of water molecules. In addition, a matrix having O–H groups could undergo two modes of hydrogen bonding; inter- and intramolecular hydrogen bonds between O–H groups [27]. Consequently, it could be concluded that incorporating 2.0 wt % CNFs in the epoxy matrix reduced its network formation process via lowering the crosslinking bonds and consequently changed the ratios of hydrogen bonding modes which lead to different geometry with different force constants and consequently shifting the wave number absorption value.

Overall, the O–H band intensity decreases and is shifted to a lower wave number. This observed progressive shift of the (υ O–H) band from about 3410 cm^−1^ toward lower wave numbers (3320 cm^−1^) can be attributed to redistribution in the arrangement of hydroxyl group association due to the different geometry caused by lowered cross-linked matrix.

To further confirm the above hypothesis, DMA testing was conducted on neat epoxy and CNFs/epoxy nanocomposites. According to the rubber elasticity theory modified by Nielsen [28], the crosslinking density was determined from the storage modulus of composites in rubbery plateau using the following equations [29,30]:

(5) $$\upsilon =\;\frac{E}{3RT}$$

where υ is the crosslinking density, *E* is the storage modulus at (*Tg* + 50), *T* is the absolute temperature of (*Tg* + 50) K and *R* is the gas constant. The molecular weight between crosslinks was given using Equation (6).

(6) $$M_{c}=\;\frac{\rho }{\upsilon }$$

where *M_c_* is molecular weight between crosslinks and *ρ* is the density of the polymer composite. The degree of crosslinking *X_link_* of the polymer composites is suggested as an inverse for the molecular weight between crosslinks in a unit volume as in Equation (7):

(7) $$X_{Link}=\;\frac{1}{M_{C}}$$

**Figure 7:**
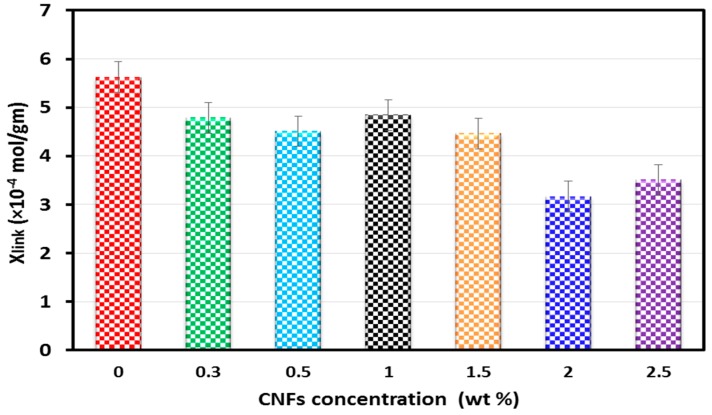
The degree of crosslinking for CNFs/epoxy nanocomposites.

The calculated degree of crosslinking *X_link_* is presented in Figure 7. The results show that incorporating 0.3, 0.5, 1.0 and 1.5 wt % CNFs in the epoxy resin slightly reduced the crosslinking by 14.8%, 19.7%, 13.8% and 20.5% respectively as can be observed in Figure 4a,b. Such a relatively low decrease in epoxy crosslinking does not have a significant negative effect on mechanical behavior of CNFs/epoxy nanocomposites due to the compensation for that decrease with the high increase in mechanical properties (e.g., modulus of elasticity) by CNFs as fibers in the matrix. On the contrary, incorporating 2.0 and 2.5 wt % CNFs in the epoxy resin reduced the crosslinking by 44% and 38% respectively owing to strong interaction between CNFs and the epoxy matrix, leading to degradation of epoxy crosslinking resulting in sharp drop in elastic modulus. The above analysis, consistent with the FTIR observations, proved that the degree of crosslinking plays a significant role in the mechanical behavior of CNFs/epoxy nanocomposites.

### 3.2. Electrical Properties of CNFs/Epoxy Nanocomposites

**Figure 8:**
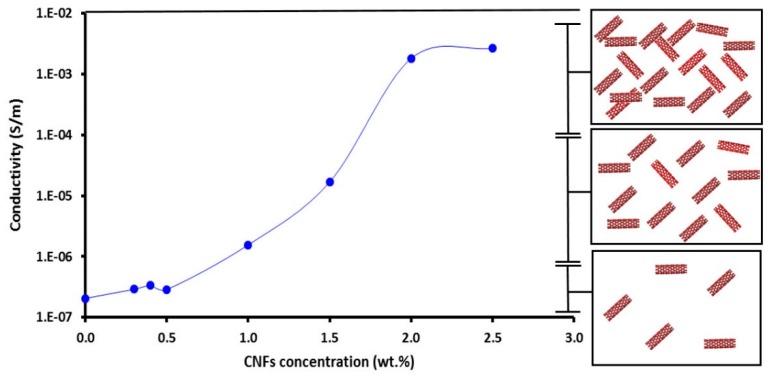
Change in electrical conductivity vs. weight percentage of CNFs in epoxy. The right sketch is a schematic describing the significance of altering CNFs content on the formation of the conductive network inside epoxy.

The epoxy matrix has inherently poor electrical conductivity. The most effective method to overcome this limitation is to add conducting fillers. Figure 8 shows the electrical conductivities (σ) for CNFs/epoxy nanocomposites at different filler loadings. The unfilled neat epoxy (at 0 wt %) is an insulator that exhibits low electrical conductivity of approximately 2.1 × 10^−7^ S/m. When CNFs were added to epoxy resin, the electrical conductivity increased. The percolation phenomenon was observed when, at low CNFs content, the conductivity of the CNFs/epoxy nanocomposites was very close to the conductivity of the epoxy matrix, because no conductive CNFs network was formed. As more CNFs were added, the fibers moved close together, and at a certain concentration (percolation threshold), a continuous conductive network of carbon nanofibers was formed and the conductivity increased by several orders of magnitude.

**Figure 9:**
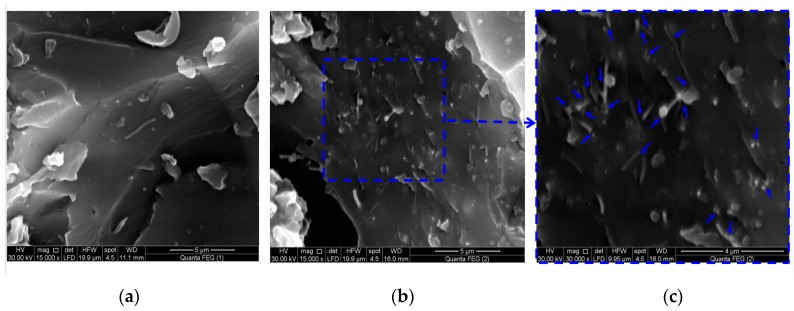
FESEM images of CNFs in the epoxy matrix; (**a**) 0.5 wt %; (**b**) 2.0 wt %; and (**c**) a close view of 2.0 wt % CNFS shows the formation of conductive network inside epoxy.

The CNFs/epoxy percolated at 1.5 wt % which was determined by the conventional method, i.e., the peak position of dlogσ/dC [31] and the electrical conductivity (σ) at 2.0 wt % is approximately 1.81 × 10^3^ S/m. The illustration in Figure 8 is proposed to explain the mechanism of the percolation phenomenon. The relatively low aspect ratio of CNFs played an important role in achieving good dispersion and resulted in a relatively high percolation limit compared with other nanofillers reported in the literature [19]. Figure 9 shows the FESEM images for 0.5 and 2.0 wt % CNFs in the epoxy matrix. The images show the absence of agglomeration of CNFs as an indication of the effective dispersion process using sonication, shear mixing and mechanical string. Figure 9c shows a close view of 2.0 wt % CNFs in epoxy with the network formation that exhibited percolation behavior. The blue arrows mark the conductive network which occurs as the CNFs are getting close to each other, creating electron paths through the matrix and thus increasing electrical conductivity.

### 3.3. Monitoring Damage Propagation of GFRP Composite under Static Tension

**Figure 10:**
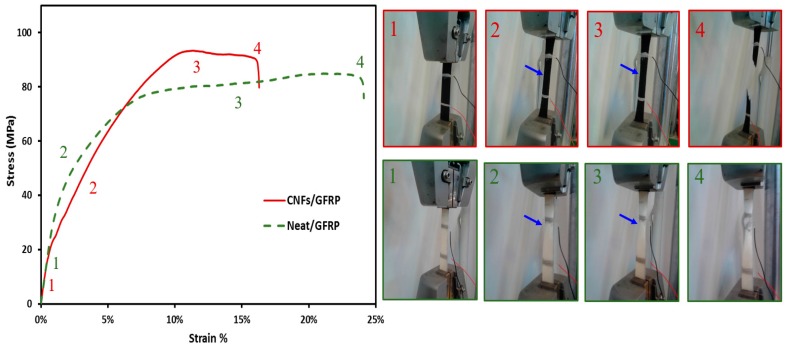
Typical stress-strain curves of neat glass fiber composite and GFRP incorporating 2.0% CNFs. Right-hand-side photos show test instances and corresponding values on stress-strain.

The stress-strain curves of GFRP composite coupons with and without 2.0 wt % CNFs under static tension are presented in Figure 10. It can be observed that the GFRP coupons show nonlinear behavior for both composites with and without CNFs. 

The source of nonlinearity is in the off-axis direction; the polymer matrix dominates the tension behavior rather than the fibers [32]. It was also observed that incorporating CNFs had no effect on the initial elastic modulus. In the off-axis direction, the composite behavior is dominated by the matrix. At low applied stress (region 1), the contribution of glass fiber shall not be neglected. The relative high stiffness of glass fiber counteracts the effect of the CNFs. When the applied stress increases (region 2), the effect of CNFs on the composite behavior starts to appear through interlaminar debonding taking place as a result of reduced fiber-matrix bond due to reduced crosslinking. This is reflected in region 2 and in the decreased stiffness of CNFs/GFRP coupons compared with GFRP composite with neat epoxy. At high applied stress levels (region 3), another effect of CNFs becomes apparent. The reduced polymer crosslinking results in softer matrix compared with neat polymer matrix. Such softening in the polymer matrix limits its ability to restrain lateral fiber movement under tension loads, thus an apparent necking like behavior takes place. This behavior is very pronounced with CNFs/GFRP coupons compared with GFRP coupons with neat epoxy. Such necking results in reduced cross-section and premature failure (region 4) at relatively lower elongation in CNFs/GFRP compared with neat GFRP. The right hand side images in Figure 10 show the deformed shape of the off-axis tensile test for both CNFs/GFRP and neat/GFRP at all four regions. The neck like feature in images 2 and 3 confirms the significant necking like behavior taking place in CNFs/GFRP coupons compared with GFRP composite with neat epoxy.

**Figure 11:**
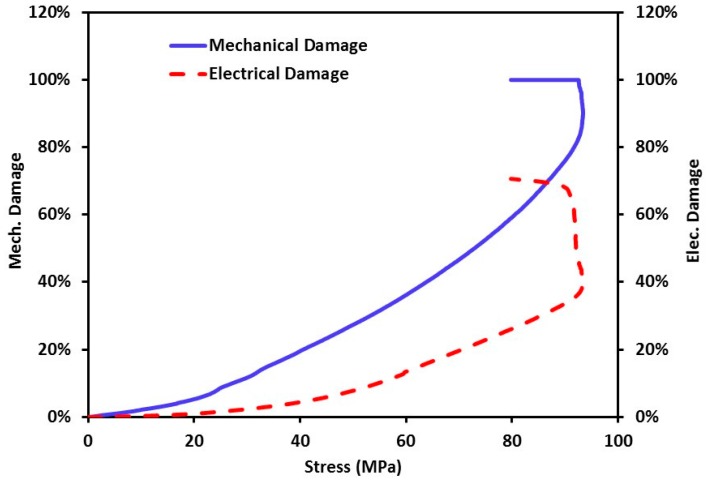
Stress-electrical damage (*D_E_*) and stress-mechanical damage (*D_M_*) for glass fiber composite incorporating CNFs under monotonically increasing static tension stress.

Figure 11 shows damage propagation in GFRP composite coupons incorporating 2.0 wt % CNFs under static tension. The figure shows a comparison between the damage metric observed using electrical conductivity monitoring and that quantified from the mechanical test using Equation (3). It is apparent that incorporating CNFs enables monitoring damage initiation and propagation with reasonable accuracy. Furthermore, both metrics increased gradually and reached a relatively flat plateau showing constant damage in GFRP at the peak stress. However, it can also be observed that the mechanical damage value was always higher than the electrical damage value for the same stress. The mechanical damage increase rate (damage propagation) is much higher for the mechanical damage metric compared with the electrical damage metric. For instance, at 40 MPa (representing about 40% of off-axis strength of GFRP), a significant level of mechanical damage metric (about 30%) is observed in the GFRP coupon compared with a very limited damage (about 5%) observed by the electrical damage metric. The difference between the mechanical and electrical damage metrics can be explained by the difference in significance of microcracks and microcrack propagation on elastic modulus and electrical conductivity. While the elastic modulus is known to be significantly affected by cracking [33], the electrical conductivity might not be influenced at the same rate by cracking as long as alternative electrically conductive paths can be found in the matrix. This means that using CNFs will provide means of monitoring damage initiation and propagation in GFRP but it might not be as sensitive to damage occurrence and propagation as mechanical damage. In essence, calibration of the two metrics might be necessary if electrical conductivity is to be used to monitor damage propagation in GFRP incorporating CNFs. Nevertheless, it is important to note that such damage initiation and propagation monitoring is not possible in neat GFRP composites due to being non-conductive.

## 4. Conclusions

The mechanical and electrical properties of epoxy incorporating CNFs were examined. The results of the electrical measurements show that CNFs can significantly improve the electrical conductivity of epoxy. It was also observed that a significant improvement in the electrical conductivity of CNFs/epoxy nanocomposite is achieved with a percolation threshold equal to 1.5 wt %. The electromechanical behavior of GFRP incorporating 2.0 wt % CNFs was then examined under monotonically increased static loading. Electromechanical measurements of GFRP coupons under off-axis static tension tests showed that electrical damage based on the change in electrical conductivity of GFRP can be correlated well with growing damage in the GFRP coupons under static tension loads. The measurements also showed that damage propagation monitoring in CNFs/GFRP using electrical conductivity has a lower sensitivity than mechanical damage propagation. This might be attributed to the pronounced effect of microcracking on mechanical behavior compared with its influence on electrical conductivity. Nevertheless, it is evident that using CNFs during fabrication of GFRP composites allows damage initiation and damage propagation in GFRP to be monitored with acceptable repeatability and resolution.
